# Supplementary material for: Continuous mark-release recapture to improve estimates of movement and survival of the African malaria mosquitoes
Source: bioRxiv. 2026 Jun 25:2026.06.24.734339. Preprint. [Version 1] doi: 10.64898/2026.06.24.734339 (PMC13320904; doi:10.64898/2026.06.24.734339)

bioRxiv preprint doi: https://doi.org/10.64898/2026.06.24.734339; this version posted June 25, 2026. The copyright holder for this preprint (which was not certified by peer review) is the author/funder. This article is a US Government work. It is not subject to copyright under 17 USC 105 and is also made available for use under a CC0 license.

Supplemental Information

Figure S1. Mosquito capture variation between zones and species. The total number of females and males collected per zone (a). The relationship between mean indoor density of males and females in each day of collection with Pearson Correlation coefficient (b). The relationships of female and male indoor density per zone to the number of houses per zone, the approximate area of each zone, and the house density per zone (c). Trend (regression) lines and correlation coefficients are shown.

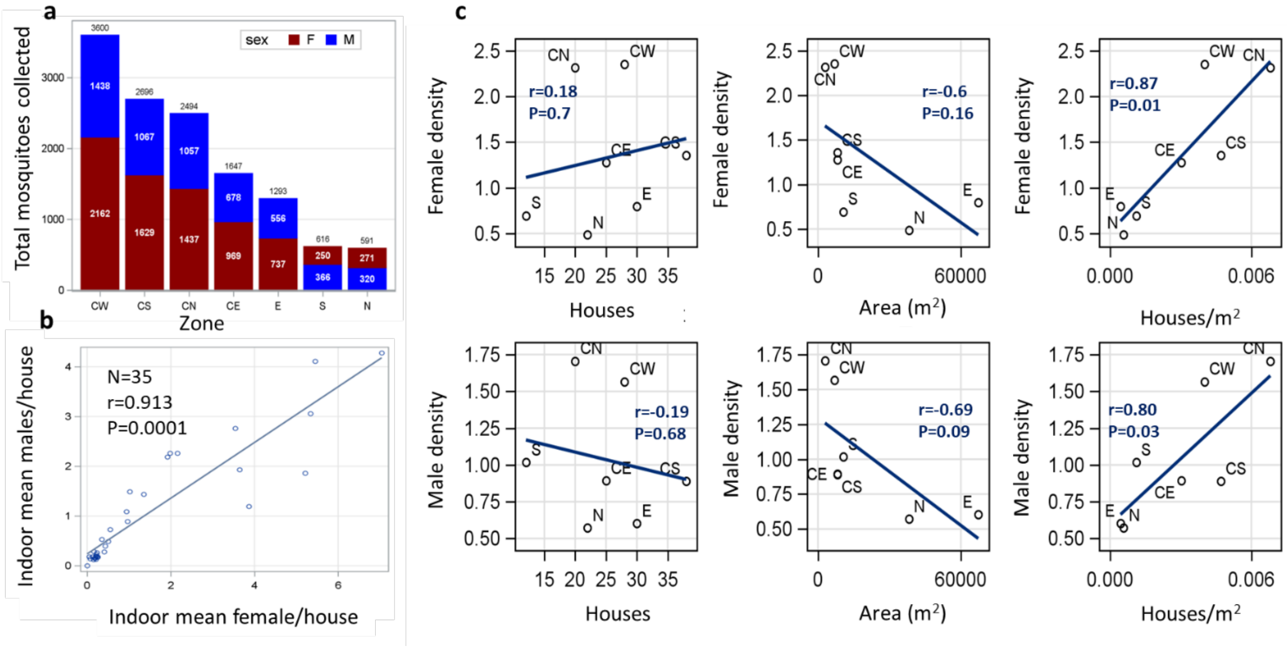

bioRxiv preprint doi: https://doi.org/10.64898/2026.06.24.734339; this version posted June 25, 2026. The copyright holder for this preprint (which was not certified by peer review) is the author/funder. This article is a US Government work. It is not subject to copyright under 17 USC 105 and is also made available for use under a CC0 license.

Figure S2. Increase in sporozoite infection rate (thorax-head infection) among recaptured females over time since release. Sample size per time point is given below each point and its time period above. Note that due to small sample size, recaptured females collected 9 or more days after release were pooled, denoted as 'Recap9+' and broken X axis. Time zero relates to the sporozoite infection rate measured at capture among dead females. Linear regression and 95%CI of the expected mean are shown with regression equation, statistical significance (P).

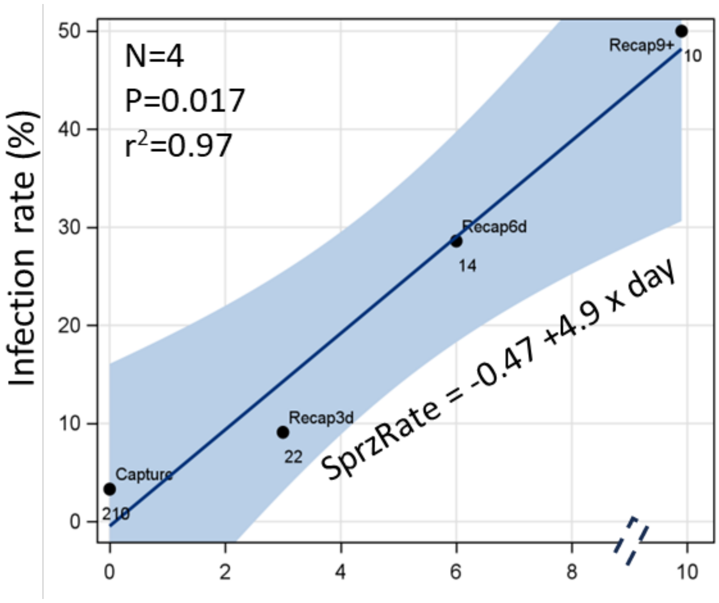

Supplement: 1 [file NIHPP2026.06.24.734339v1-supplement-1.pdf]
